# Bursal Cysts in Connection with the Knee-Joint

**Published:** 1909-02-27

**Authors:** 


### Surgery.

### BURSAL CYSTS IN CONNECTION WITH THE KNEE-JOINT.

Bursal cysts in connection with joints are usually all classed as "Baker's cysts," so named after Mr. Morrant Baker, of St. Bartholomew's hospital, who first described them. A true Baker's cyst consists of a hernial protrusion of the synovial membrane through the fibrous capsule of the joint and is, therefore, somewhat analogous to a ganglion, which is a similar protrusion through the sheath of a tendon.

Thus a Baker's cyst is rarely, if ever, a clinical entity in itself. It should be regarded, rather, merely as a symptom of disease of the joint in whose neighbourhood it appears. The disease of the joint causes an effusion into the synovial cavity and increases the intra-articular pressure, and the extra fluid is forced out into an adventitious cyst, which acts, as it were, as a safety-valve.

But most joints have in connection with them bursæ, which are placed in the line of the peri-articular tendons to facilitate their action. These are, morphologically speaking, processes of the adjacent synovial membrane, but they should be completely cut off from it. Sometimes, however, they are still connected with the joint cavity by a slender but pervious stalk, and they may then become cysts without any actual disease of the joint itself, or a very slight increase of the normal pressure; a simple chronic synovitis resulting from injury may be sufficient to distend them. The knee-joint is particularly well provided with peri-articular bursæ. In front there is the prepatellar bursa and another one deep to the ligamentum patellæ. There is one on the inner side under the combined tendon of insertion of the gracilis, semi-tendinosus and sartorius, and on the outer side between the biceps and the external lateral ligament. Behind there is one in each of the following situations: under the insertion of the semi-membranosus, under the tendon of the popliteus, and under each head of origin of the gastrocnemius.

Clinically cysts may be found in any of these situations: they are rare, however, except in front of or behind the ligamentum patellæ, beneath the quadriceps extensor muscle, and in the popliteal space. This is the commonest situation for them, and it would seem that the bursa under the semi-membranosus is the one most commonly affected.

There are certain classical signs on which reliance may be placed in making a diagnosis. Thus the swelling is formed slowly, and is painless, the patient complaining, in most cases, only that its presence impedes full use of the joint. Manipulation is not resented and the swelling either feels elastic or definitely fluctuates. In the latter case it is often possible to reduce the contained fluid into the synovial cavity when the joint is flexed. The ease with which this can be accomplished varies directly with the size of the communication between the bursa and the joint. If this is free, pressure on the cyst may be felt to distend the joint. Sometimes if the cyst lies in loose areolar tissue, a pedicle may be felt leading towards the joint.

In such a case the diagnosis is comparatively easy, but it sometimes happens that the cyst has wandered away from the joint although still connected with it by a pervious pedicle. In the case of the knee these wandering cysts travel down between the muscle planes in the calf of the leg, and one case at least has been recorded of a bursa in connection with the knee-joint presented in the vicinity of the external malleolus. It is important to bear this in mind, because in operating on such a condition it is essential to open it with all the circumspection and antiseptic precaution that one would use in opening the knee-joint itself: for however long and devious the track which connects it with the joint, infection readily spreads along it, and if the technique is faulty acute suppurative arthritis of the knee-joint may ensue—a surgical disaster of the first magnitude.

It has been said above that the presence of these cysts must be regarded as presumptive evidence of disease of the joint itself, and the conditions which one usually finds associated with them are osteo-arthritis and tuberculosis—usually the former, since it more often produces hydrops articuli than does tubercle. This is a fact which necessarily alters the prognosis, because the removal of the cyst does not cure the condition as it does in the so-called idiopathic Baker's cyst. But it does not, in our opinion, contra-indicate surgical interference. Some twelve months ago the writer had under his care a boy of 15 with an oval fluctuating swelling on the inner side of the popliteal space, the size of a goose's egg, which had existed for three months. It was slightly painful and tender and impeded full flexion of the joint. A vertical incision was made over the swelling and it was dissected away from its surroundings. It was found to be an enlarged semi-membranosus bursa. Its connection with the joint was ligatured, and it was removed. It was opened afterwards and found to be full of caseating tuberculous material, and the walls of the cyst, on microscopic examination, were seen to be typically tuberculous, with many giant-cell systems. The boy made an uninterrupted recovery and has been kept under observation since. Up to the time of writing, one year after the operation, he has developed no sign of tuberculosis of the knee-joint. It must be supposed that the disease of the bursa was here a primary infection and that the joint itself escaped.

The best treatment of these bursal cysts is to remove them by an open operation. Attempts to get rid of them by blistering, pressure, or the injection of irritants are antiquated methods and may do more harm than good. But the operation is not always easy. The cyst wall is often thin, and if the cyst is opened before the capsule is isolated the difficulty will be increased. It is important to get into the right layer in isolating the cyst. To do this the fibrous laminæ covering it must be dissected off until the true capsule of the cyst, which is recognised by its translucent appearance, comes into view. If it has a pedicle, this is ligatured, but if, as sometimes happens, its base is broad, it must be cut away and the edges of the rent left united with catgut. The wound is then sewn up in the ordinary way.

